# Supplementary material for: Association between hypertriglyceridemic waist phenotype and hypogonadism in Taiwanese adult men
Source: PLoS One. 2022 Mar 23;17(3):e0265629. doi: 10.1371/journal.pone.0265629 (PMC8942218; doi:10.1371/journal.pone.0265629)
Supplement: Attachment — Submitted filename: Point-by-point response.docx [file pone.0265629.s001.docx]

**Responses to reviewers’ comments on “Association between hypertriglyceridemic waist phenotype and hypogonadism in Taiwanese adult men”**

(**Submission ID: PONE-D-21-22955**)

We appreciate the reviewers’ comments. Our point-by-point responses are provided below (Line and page numbers are shown for convenience, and the revised material is indicated in red text in the revised manuscript).

**Reviewer #1:**

Abstract:

lines 118-120 is not clear what the p value is referring to. In the text authors report that “there was a statistically significant difference between the phenotypes (NWNT, EW, HTG, and HTGW) and hypogonadism (p < 0.001).”, but from table 2 p value seems about the difference between group with and without hypogonadism. However, it appears from numbers displayed in table 2 that there is a higher prevalence of HTGW and a lower prevalence of NWNT in those with hypogonadism than in those without hypogonadism, but among those with hypogonadism it seems that the prevalence of HTGW is similar to that of NWNT. Nevertheless these are only impressions from numbers because pairwise p values are not reported. The same for the analyses showed in table 3 and lines 126-132.
**Response: page 9, lines 131-134, 138-142**

We thank the reviewer for their comment. With reference to the literature (Okada, 2020; Chien et al., 2020), we have adjusted the contents of Tables 2 and 3 to clearly express the correlation between phenotypes and hypogonadism. The *p*-value refers to the use of a chi-square test. We have modified the results section as follows:

The overall prevalence of hypogonadism among all participants was 10.63%. As shown in Table 2, the distribution of phenotypes (NWNT, EW, HTG, and HTGW) was significantly different between participants with and without hypogonadism (*p* <0.001). Participants without hypogonadism were more likely to have the NWNT phenotype than those with hypogonadism.

The prevalence of each phenotype in those with and without hypogonadism in each age group is shown in Table 3. In age group 1, participants with hypogonadism were significantly more likely to be HTGW than those without (*p* <0.001). In age group 2, the NWNT phenotype was significantly more prevalent among those with hypogonadism than those without (*p* <0.001). However, there were no significant differences in phenotypes between those with and without hypogonadism in age group 3.

**References**

Chien KH, Huang KH, Chung CH, Hsieh YH, Liang CM, Chang YH, et al. The impact of diabetes mellitus medication on the incidence of endogenous endophthalmitis. PLoS One 2020;15: e0227442.

Okada C, Takimoto H. (Development of a screening method for determining sodium intake based on the Dietary Reference Intakes for Japanese, 2020: a cross-sectional analysis of the National Health and Nutrition Survey, Japan. PLoS One 2020;15: e0235749.

1. Authors derives from chi-square test the correlation between these subgroups, but chi-square actually report information about the difference in distribution and not about correlation. Consequently, the logistic regression analyses is not really supported from previous analyses and implies a causal relationship that is not justified by the presented data.

   I suggest analysing the variables as continuous variables and using appropriate tests to highlight possible correlations and then detect the presence of potential hypogonadism independent determinants through multivariate regression analysis.

**Response:** **page 11, lines 147-155**

We thank the reviewer for their comment. We conducted a linear regression analysis, the results of which are shown in the table below (table not in manuscript). The effects of phenotypes on hypogonadism were found to be similar using this method. We analyzed hypogonadism as a binary dependent variable according to the approach established in the prevalence of hypogonadism study by Mulligan et al. (2006). However, the total testosterone level distribution was found to be abnormal. Therefore, we used the results of the logistic regression model.

**References**

Mulligan T., Frick MF, Zuraw QC, Stemhagen A, & McWhirter C. Prevalence of hypogonadism in males aged at least 45 years: The HIM study. Int J Clin Pract 2006;60: 762-769.

**Table 0. Results of linear regression analysis**

|  | **Age < 50 years**  **(n = 4135, age group 1)** | | | **Age 50–64 years**  **(n = 1958, age group 2)** | | | **Age ≥ 65 years**  **(n = 349, age group 3)** | | |
| --- | --- | --- | --- | --- | --- | --- | --- | --- | --- |
|  | **β** | **p** | **95% CI** | **β** | **p** | **95% CI** | **β** | **p** | **95% CI** |
| crude |  |  |  |  |  |  |  |  |  |
| NWNT | Ref. |  |  | Ref. |  |  | Ref. |  |  |
| EW | -121.09 | <0.001*** | -139.60, -105.59 | -133.49 | <0.001*** | -161.71, -105.28 | -126.84 | <0.001*** | -191.30, -62.39 |
| HTG | -103.76 | <0.001*** | -118.99, -88.53 | -121.59 | <0.001*** | -148.30, -94.88 | -99.43 | 0.014* | -178.36, -20.49 |
| HTGW | -172.82 | <0.001*** | -191.11, -154.53 | -161.99 | <0.001*** | -193.51, -130.46 | -170.59 | <0.001*** | -247.47, -93.71 |
| adjusted |  |  |  |  |  |  |  |  |  |
| NWNT | Ref. |  |  | Ref. |  |  | Ref. |  |  |
| EW | -18.01 | 0.098 | -39.37, 3.35 | -39.10 | 0.025* | -73.22, -4.98 | -32.23 | 0.429 | -112.30, 47.83 |
| HTG | -75.68 | <0.001*** | -92.59, -58.77 | -95.89 | <0.001*** | -125.30, -66.47 | -71.19 | 0.107 | -157.84, 15.46 |
| HTGW | -62.16 | <0.001*** | -84.88, -39.43 | -66.25 | <0.001*** | -104.72, -27.77 | -96.32 | 0.046* | -190.89, -1.75 |

CI, confidence interval; EW, enlarged waist and normal triglyceride levels; HTG, hypertriglyceridemia and normal waist; HTGW, Hypertriglyceridemia and waist circumference ≥90 cm; NWNT, normal waist and normal triglyceride levels.

**Reviewer #2:**

1. Abstract: The Abstract is confused, results should be presented briefly, and the Authors should provide at least two rows of background/introduction.

**Response:** **page 2, lines 26-29**

We thank the reviewer for their comment. We have adopted the reviewer’s suggestion and added the following to the abstract.

The hypertriglyceridemic enlarged waist (HTGW) phenotype is a tool for predicting abnormalities of cardiovascular metabolism. However, the relationship between the HTGW phenotype and hypogonadism remains undetermined.

2. Materials and methods:
   - Please specify why the Authors choose total testosterone instead free testosterone?

**Response: page 13, lines 208-212**

We thank the reviewer for their comment. We used the health examination database in this study, from which free testosterone data could not be obtained. We have added the following passage to the limitations subsection.

Second, we evaluated testosterone deficiency based on a single measurement of total testosterone level because the free testosterone data could not be obtained from the health examination database used in this study. We hope to conduct another retrospective study in the future to investigate the relationship between the HTGW phenotype and free testosterone.

3. - Authors should provide more data about the causes of low testosterone levels, e.g., injury, trauma, infections, or tumors of the testes? Medications or radiations exposure? Liver diseases? Were these cases of hypogonadism primary or secondary? Did they perform any other tests to investigate the dysfunction of the pituitary gland? Etc. These data are essential to exclude the causality of presented results.

**Response: Figure 2 and page 11, lines 147-155**

We thank the reviewer for their comment. With reference to the literature (Laaksonen et al., 2005; Gasevic et al., 2014), we have included smoking status, cholesterol, HDL, and LDL in our analysis, and have revised the “Results” section as shown below. As the health database does not collect specific measures of nicotine intake, it was not possible to analyze the tobacco use dosages.

As shown in Figure 2, we investigated the effects of the four phenotypes on the risk of hypogonadism. In age group 1, participants with the HTGW (odds ratio [OR], 1.980; 95% confidence interval [CI], 1.354-2.896) and HTG phenotype (OR, 1.803; 95% CI, 1.297-2.505) had higher risks of hypogonadism than those with the NWNT phenotype, after adjustment for BMI and FBG levels. In age group 2, participants with the HTGW (OR, 1.873; 95% CI, 1.099-3.193) and HTG (OR, 2.098; 95% CI, 1.334-3.298) phenotypes also had higher risks of hypogonadism than those with NWNT after adjustment for BMI, FBG level, cholesterol level, HDL level, LDL level, and smoking status. However, no relationship was observed between the phenotypes and hypogonadism in age group 3.

Odds ratios for hypogonadism in age group 1 (aged <50 years)

Odds ratios for hypogonadism in age group 2 (aged 50-64 years)

Odds ratios for hypogonadism in age group 3 (aged ≧65 years)

**Figure 2**. Odds ratio for hypogonadism stratified by age group.

Adjusted OR were adjusted for body mass index, fasting blood glucose levels, cholesterol levels, high-density lipoprotein levels, low-density lipoprotein levels, and smoking status.

NWNT, normal waist circumference and normal triglyceride levels; HTG, hypertriglyceridemia and normal waist circumference; EW, enlarged waist and normal triglyceride levels; HTGW, hypertriglyceridemia and waist circumference ≥90 cm.

**References**

Gasevic D, Carlsson AC, Lesser IA, et al. The association between “hypertriglyceridemic waist” and sub-clinical atherosclerosis in a multiethnic population: A cross-sectional study. Lipids Health Dis 2014;13: 38.

Laaksonen DE, Niskanen L, Punnonen K, Nyyssönen K, Tuomainen TP, Valkonen, VP, Salonen JT. The metabolic syndrome and smoking in relation to hypogonadism in middle-aged men: A prospective cohort study. J Clin Endocr Metab. 2005;90: 712-719.

4. Results: data are not clearly exposed, e.g., lines 119-120 they affirm that “there was a statistically significant difference between the phenotypes (NWNT, EW, HTG, and HTGW) and hypogonadism (p < 0.001)” – I suggest to re-phrase as “the percentage of EW, HTG, and HTGW was significantly higher in the group of patients with hypogonadism compared with those with normal testosterone values” and to add the exact p values for each group. In table 2 and table 3, what the p value referring to?

**Response: page 9, lines 131-134, 138-142**

We thank the reviewer for their comment. With reference to the literature (Chien et al., 2020; Okada & Takimoto, 2020), we adjusted Tables 2 and 3 to more clearly express the correlations between phenotypes and hypogonadism. The *p*-value refers to the use of the chi-square test. We have modified the results section as follows:

The overall prevalence of hypogonadism in all participants was 10.63%. As shown in Table 2, the distribution of phenotypes (NWNT, EW, HTG, and HTGW) was significantly different between participants with and without hypogonadism (*p* <0.001). Participants without hypogonadism were more likely to have the NWNT phenotype than those with hypogonadism.

Table 3 shows the prevalence of hypogonadism in the different age groups. In age group 1, participants with hypogonadism were more likely to have the HTGW (*p* <0.001) phenotype. In age group 2, the NWNT phenotype was more prevalent among participants with hypogonadism (*p* <0.001). However, there was no significant relationship between hypogonadism and phenotypes in age group 3.

**References**

Chien KH, Huang KH, Chung CH, Hsieh YH, Liang CM, Chang YH, et al. The impact of diabetes mellitus medication on the incidence of endogenous endophthalmitis. PLoS One 2020;15: e0227442.

Okada C, Takimoto H. Development of a screening method for determining sodium intake based on the Dietary Reference Intakes for Japanese, 2020: A cross-sectional analysis of the National Health and Nutrition Survey, Japan. PLoS One 2020;15: e0235749.

4. Discussion: I suggest including a more in-depth explanation of the possible pathogenic mechanisms involved in the relationship between metabolic disorders, such as HTGW, and low testosterone levels. Moreover, personally, I can hardly understand if hypogonadism is the consequence or a potential cause of HTGW.

**Response: page 12, lines 173-185**

We thank the reviewer for their comment. We have adopted their suggestion and added the following passage to the discussion.

Several studies have shown that visceral obesity in men is associated with low testosterone levels, male infertility, and erectile dysfunction [24–28]. In animal experiments, weight loss was associated with a rise in levels of testosterone, free testosterone (FT), and sex hormone-binding globulin (SHBG), whereas weight gain was associated with a fall in these levels [29]. However, hypogonadism may induce visceral obesity. In a castrated animal model, weight significantly increased, especially in terms of visceral fat accumulation [30]. This implies that hypogonadism has a bidirectional relationship with obesity. Among the components of metabolic syndrome, it has the strongest association with visceral obesity [31, 32]. Indeed, male hypogonadism can result from complex interactions among lifestyle, metabolic health, and genetic background. Endocrine disruption caused by metabolic diseases can trigger the onset of hypogonadism, although the underlying mechanisms are not fully understood [33]. Metabolic disorders may modify the hypothalamic-pituitary-gonadal (HPA) axis by periodically suppressing one or more of its components; whereas, in permanent forms of hypogonadism, one or more of the HPA axis components irrevocably loses functionality [34].

**References**

24. Tchernof A, Després JP. Pathophysiology of human visceral obesity: An update. Physiol Rev. 2013;93: 359-404.
25. Amjad S, Baig M, Zahid N, Tariq S, Rehman R. Association between leptin, obesity, hormonal interplay, and male infertility. Andrologia 2019;51: e13147.
26. Huang IS, Mazur DJ, Kahn BE, Keeter MK, Desai AS, Lewis K, et al. Risk factors for hypogonadism in young men with erectile dysfunction. J Chin Med Assoc. 2019;82: 477-481.
27. Turan E, Öztekin Ü. Relationship between visceral adiposity index and male infertility. Andrologia 2020;52: e13548.
28. Sun K, Wang C, Lao G, Lin D, Huang C, Li N, et al. Lipid accumulation product and late-onset hypogonadism in middle-aged and elderly men: Results from a cross-sectional study in China. BMJ Open 2020;10: e033991.
29. Georgiev IP, Georgieva TM, Ivanov V, Dimitrova S, Kanelov I, Vlaykova T, et al. Effects of castration-induced visceral obesity and antioxidant treatment on lipid profile and insulin sensitivity in New Zealand white rabbits. Res Vet Sci. 2011;90: 196-204.
30. Camacho EM, Huhtaniemi IT, O'Neill TW, Finn JD, Pye SR, Lee DM, et al. Age-associated changes in hypothalamic-pituitary-testicular function in middle-aged and older men are modified by weight change and lifestyle factors: Longitudinal results from the European Male Ageing Study. Eur J Endocrinol. 2013;168: 445-55.
31. Crisóstomo L, Pereira SC, Monteiro MP, Raposo JF, Oliveira PF, Alves MG. Lifestyle, metabolic disorders and male hypogonadism – A one-way ticket? Mol Cell Endocrinol. 2020;516: 110945.
32. Brand JS, Rovers MM, Yeap BB, Schneider HJ, Tuomainen TP, Haring R, et al. Testosterone, sex hormone-binding globulin, and the metabolic syndrome in men: An individual participant data meta-analysis of observational studies. PLoS One. 2014;9: e100409.
33. Wang N, Zhai H, Han B, Li Q, Chen Y, Chen Y, et al. Visceral fat dysfunction is positively associated with hypogonadism in Chinese men. Sci Rep. 2016;6: 19844.
34. Lunenfeld B, Mskhalaya G, Zitzmann M, Arver S, Kalinchenko S, Tishova Y, et al. Recommendations on the diagnosis, treatment, and monitoring of hypogonadism in men. The Aging Male. 2015;18: 5-15.
