# Supplementary material for: Association between hypertriglyceridemic waist phenotype and hypogonadism in Taiwanese adult men
Source: PLoS One. 2022 Mar 23;17(3):e0265629. doi: 10.1371/journal.pone.0265629 (PMC8942218; doi:10.1371/journal.pone.0265629)
Supplement: Attachment — Submitted filename: Point-by-point response_1110209.docx [file pone.0265629.s002.docx]

**Responses to reviewers’ comments on “Association between hypertriglyceridemic waist phenotype and hypogonadism in Taiwanese adult men”**

(**Submission ID: PONE-D-21-22955**)

We appreciate the reviewers’ comments. Our point-by-point responses are provided below (Line and page numbers are shown for convenience, and the revised material is indicated in red text in the revised manuscript).

**Reviewer #2:**

The new version of the manuscript seems to be now clearer and more comprehensible. In particular, the aim of the study is precise and the results are more intelligible. The Authors improved their text addressing to nearly all comments reported by Reviewers. I have one suggestion to improve even more the quality of this manuscript; in the Study Population section, I would specify that the population under the study was free from any pathological condition that affects the testes function (e.g. injury, trauma, infections, or tumors, endocrinological disorders, etc...).
**Response: page 4, lines 81-82**

**page 13, lines 211-216**

We thank the reviewer for comment. We have modified the excluded criteria and added the following contents in the limitation subsection.

Study population

Subsequently, we excluded participants with prostate cancer and insufficient data on serum testosterone level.

Limitations

This study has several limitations. First, this cross-sectional study could not clarify the causality between the HTGW phenotype and hypogonadism in the Asian population. Further studies with a prospective design are necessary to verify our findings in different ethnic populations. Second, we evaluated testosterone deficiency based on a single measurement of total testosterone level because the FT data could not be obtained from the health examination database used in this study. Furthermore, some pathological conditions that may affect testes function (eg injury, trauma, infection or tumor, endocrine disease, etc.) were not able to include in our data. We hope to conduct another retrospective study in the future to investigate the relationship between the HTGW phenotype and FT without previous pathological conditions. Third, this study did not use imaging technologies such as computed tomography and magnetic resonance imaging to evaluate the visceral fat content of the participants, so we could not understand the relationship between visceral fat and testosterone level. Additional studies are needed to examine this relationship.
